# Anti-Osteoclastic Activity of *Artemisia capillaris* Thunb. Extract Depends upon Attenuation of Osteoclast Differentiation and Bone Resorption-Associated Acidification Due to Chlorogenic Acid, Hyperoside, and Scoparone

**DOI:** 10.3390/ijms18020322

**Published:** 2017-02-04

**Authors:** Sang-Hyun Lee, Jung-Yun Lee, Young-In Kwon, Hae-Dong Jang

**Affiliations:** Department of Food and Nutrition, Hannam University, Daejeon 34430, Korea; ggamannom03@nate.com (S.-H.L.); seembeeks@hanmail.net (J.-Y.L.); youngk@hnu.kr (Y.-I.K.)

**Keywords:** *Artemisia capillaris*, osteoclast differentiation, bone resorption, acidification

## Abstract

The present study attempts to elucidate the anti-osteoporotic activity of *Artemisia capillaris* Thunb. in the form of anti-osteoclastic effect and responsible bioactive compounds. The contents of chlorogenic acid, caffeic acid, hyperoside, isoquercitrin, isochlorogenic acid A, and scoparone in *Artemisia capillaris* hydroethanolic extract (ACHE) were 38.53, 0.52, 4.07, 3.03, 13.90, and 6.59 mg/g, respectively. ACHE diminished osteoclast differentiation and bone resorption due to chlorogenic acid, hyperoside, and scoparone. In addition, ACHE attenuated acidification as well as reducing tumor necrosis factor receptor-associated factor 6 (TRAF6) expression and its association with vacuolar H^+^-adenosine triphosphatase (V-ATPase). Furthermore, chlorogenic acid, hyperoside, and scoparone from *A. capillaris* abrogated the association of V-ATPase with TRAF6, suggesting that the blockage of bone resorption by *A. capillaris* was partially mediated by reducing acidification through down-regulating interaction of V-ATPase with TRAF6 due to scoparone as well as chlorogenic acid and hyperoside. These results imply that the anti-osteoclastic effect of *A. capillaris* through down-regulating osteoclast differentiation and bone resorption may contribute to its anti-osteoporotic effect.

## 1. Introduction

The bone remodeling cycle is an elaborately controlled process of bone metabolism, in which osteoclasts resorb the mineralized matrix and osteoblasts form new bone matrix [1]. Accordingly, bone mass depends upon an orchestrated balance between osteoclastic bone resorption and osteoblastic bone formation. Osteoclast differentiation from precursors and tartrate-resistant acid phosphatase (TRAP) activity belong to essential factors for bone resorption by osteoclasts. Osteoblast differentiation and proliferation, alkaline phosphatase activity, and type I collagen synthesis are required for bone formation by osteoblasts [2]. However, the imbalance between bone resorption and bone formation can cause metabolic bone diseases such as osteoporosis and osteopetrosis [3].

Osteoclasts, bone-resorbing multinucleated cells, are differentiated by the fusion of their mononuclear precursors, monocytes and macrophages, which is triggered by the receptor activator of nuclear factor-κB ligand (RANKL) produced by osteoblasts [4,5,6]. Bone resorption requires that osteoclasts have the ability to produce protons since an acidic pH is required for the solubilization of the alkaline salts of bone matrix and the digestion of bone matrix by acid enzymes secreted by osteoclasts [7]. This acidification is due to vacuolar H^+^-adenosine triphosphatase (V-ATPase) localized on the ruffled border of osteoclasts, which hydrolyzes ATP to produce protons and extrudes them into extracellular space. It was reported that the interaction of V-ATPase with tumor necrosis factor receptor-associated factor 6 (TRAF6) is required for its activation [8].

*Artemisia capillaris* Thunb. belongs to the genus *Artemisia* and has been widely used as an edible herbal medicine in Asian countries. Many studies have reported that *A. capillaris* extracts or its bioactive compounds such as caffeoylquinic acids, flavonoids, and coumarins [9,10,11,12] have various biological functions including antioxidant activity [13,14], anti-inflammatory activity [15], anti-microbiological activity [16], anti-tumor activity [17], cytoprotective effects [14], hepatoprotective effects [18,19], and anti-osteoporosis effects [20]. The recent study demonstrated that the water extract of *A. capillaris* suppressed RANKL-induced osteoclast differentiation from bone marrow macrophages and bone resorption by the attenuated expression of biomarkers including cathepsin K and ATPase V(0) domain **(**ATPv0d2) [20]. Unfortunately, there is no systematic study on the anti-osteoclastic effect of bioactive compounds of *A. capillaris* through the down-regulation of acidification.

The objective of the present study was to investigate the anti-osteoclastic effects of *A. capillaris* and responsible bioactive compounds using an osteoclastic cells model for developing functional food in the treatment of bone diseases caused by bone loss.

## 2. Results

### 2.1. The Content of Six Marker Compounds in Artemisia capillaris Hydroethanolic Extract (ACHE)

**Figure 1:**
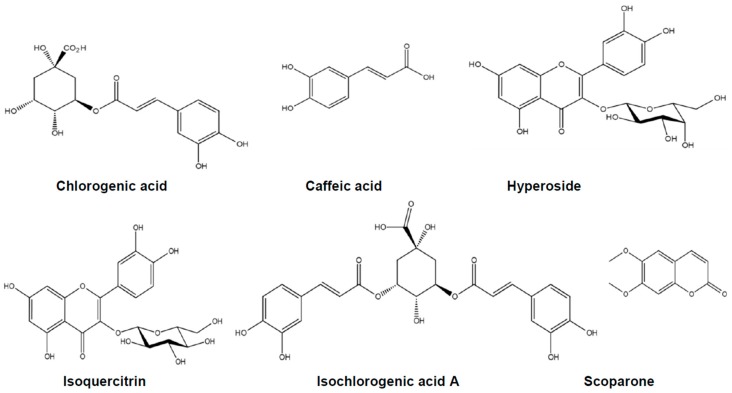
Chemical structures of six compounds.

**Figure 2:**
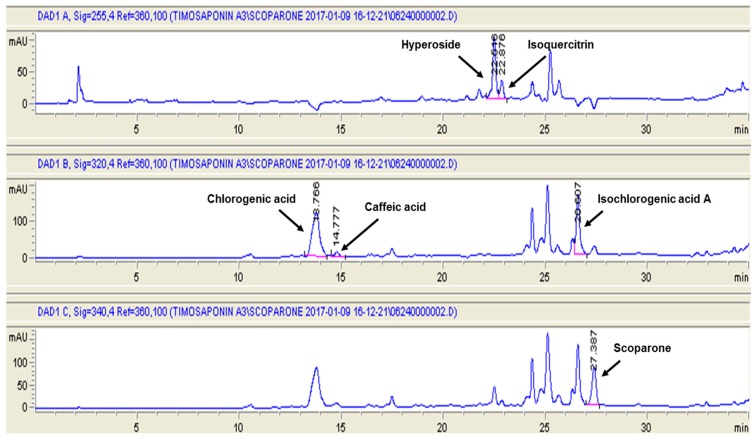
High performance liquid chromatography (HPLC) chromatograms of ethanol extract of *Artemisia capillaris*. The detection wavelength was set at 255 nm for hyperoside and isoquercitrin, at 320 nm for chlorogenic acid, caffeic acid, and isochlorogenic acid, and at 340 nm for scoparone.

Chlorogenic acid, caffeic acid, hyperoside, isoquercitrin, isochlorogenic acid A, and scoparone demonstrated in Figure 1 have been reported as six important constituents of *A. capillaris* [11]. Thus, they were identified by the retention time and ultraviolet (UV) spectrum data of standard substances and their contents were determined by straight calibration with a standard curve. As shown in Figure 2, the retention time of chlorogenic acid, caffeic acid, hyperoside, isoquercitrin, isochlorogenic acid A, and scoparone were 13.78, 14.78, 22.52, 22.88, 26.61, and 27.39 min, respectively. The content of chlorogenic acid, caffeic acid, hyperoside, isoquercitrin, isochlorogenic acid A, and scoparone in ACHE were 38.53, 0.52, 4.07, 3.03, 13.90, and 6.59 mg/g, respectively (Table 1), indicating that chlorogenic acid and isochlorogenic acid A were the major components in ACHE. This result appear to be consistent with previous study in which the content of isochlorogenic acid A was 43.14 mg/g followed by chlorogenic acid (21.06 mg/g), hyperoside (8.44 mg/g), and scoparone (5.56 mg/g) [11].

### 2.2. Suppressive Effect of ACHE and Its Six Marker Compounds on Osteoclast Differentiation

**Figure 3:**
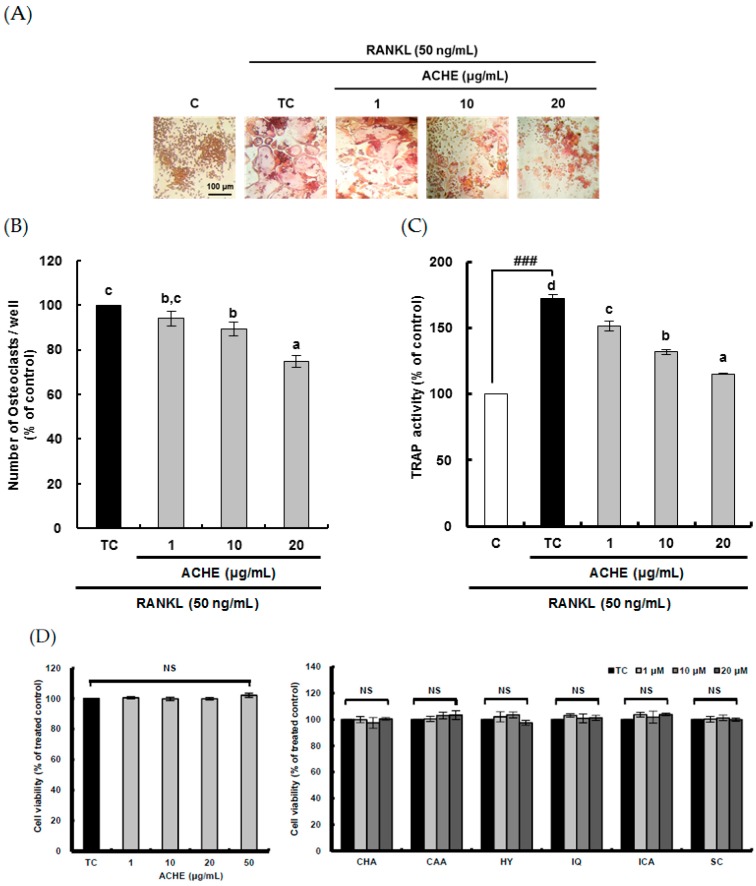

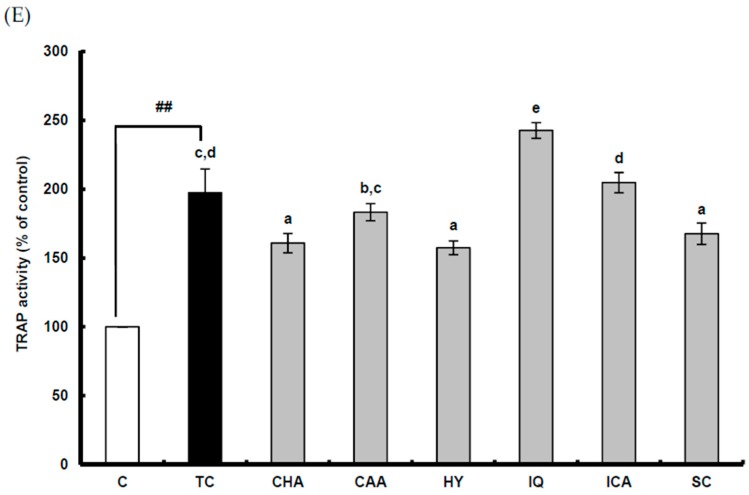
Inhibitory effect of hydroethanolic extract of *A. capillaris* and its six marker compounds on osteoclast differentiation. (**A**) The visualized tartrate-resistant acid phosphatase (TRAP)-positive multinucleated osteoclasts with *A. capillaris* hydroethanolic extract (ACHE) treatment; (**B**,**C**) The counted number and TRAP activity of TRAP-positive multinucleated osteoclasts with ACHE treatment; (**D**) The cytotoxicity of *A. capillaris* and its six marker compounds; (**E**) The TRAP activity of multinucleated osteoclasts with six marker compounds treatment. RAW 264.7 cells were exposed to receptor activator of nuclear factor-κB ligand (RANKL; 50 ng/mL) for 5 days in the absence and presence of ACHE. After 5 days in culture, the cells were fixed and stained using a leukocyte acid phosphatase kit. TRAP-positive multinucleated osteoclasts were visualized at 200-fold magnification under light microscopy. TRAP-positive multi-nucleated osteoclasts were counted and TRAP activity was measured at λ = 405 nm. Data are expressed as percentages of the value of cells treated with RANKL (means ± standard deviations, SD, *n* = 3). Data are expressed as percentages of the values of untreated cells (means ± standard deviations, *n* = 3). Different corresponding letters indicate significant differences at *p* < 0.05 by Duncan’s test. ^##^ *p* < 0.01, ^###^ *p* < 0.001 vs. C. C: control, which was not treated; TC: treated control, which was treated with RANKL; CHA: chlorogenic acid; CAA: caffeic acid; HY: hyperoside; IQ: isoquercitrin; ICA: isochlorogenic acid; SC: scoparone; NS: not significant.

To study the anti-osteoporosis effect of ACHE through the attenuated bone resorption by osteoclasts, the suppressive effects of ACHE on RANKL-induced osteoclast differentiation were analyzed. RANKL, which is required for the differentiation of monocytes/macrophages into osteoclasts, was used as an inducer of osteoclast differentiation from RAW 264.7 cells [18]. RANKL (50 ng/mL) induced TRAP-positive osteoclast formation from pre-osteoclastic cells, which was visualized by light microphotography (Figure 3A). However, ACHE at 1–20 μg/mL dose-dependently attenuated the number of osteoclasts (Figure 3B). In addition, ACHE at 1–20 μg/mL dose-dependently diminished the TRAP activity of osteoclasts which was significantly (*p* < 0.001) enhanced up to 172.1% by RANKL treatment compared to the control group (Figure 3C). To exclude the possibility that the inhibitory effect was due to the decreased viability and/or the proliferation of the pre-osteoclastic cells, cell cytotoxicity was checked. The results showed that ACHE did not affect cell viability at the doses that effectively suppressed osteoclast differentiation (Figure 2D).

To determine which of the six marker compounds of ACE contributed to inhibitory activity of ACHE in RANKL-induced osteoclast differentiation, TRAP activity was measured at the concentration of 10 μM at which scoparone moderately inhibited RANKL-induced osteoclast differentiation in our previous study [21]; the cell cytotoxicity of six compounds was not found (Figure 3D). The potent inhibitory effect on TRAP activity was observed in chlorogenic acid, hyperoside, and scoparone as compared with caffeic acid, isoquercitrin, and isochlorogenic acid at 10 μM (Figure 3E). On the other hand, isoquercitrin displayed the pro-osteoclastic effect in this experiment. Thus, this result suggests that the inhibitory effect of ACHE on RANKL-induced osteoclast differentiation may be attributed to three compounds such as chlorogenic acid, hyperoside, and scoparone.

### 2.3. Inhibitory Effect of ACHE and Three Bioactive Compounds on Bone Resorption of Osteoclast

**Figure 4:**
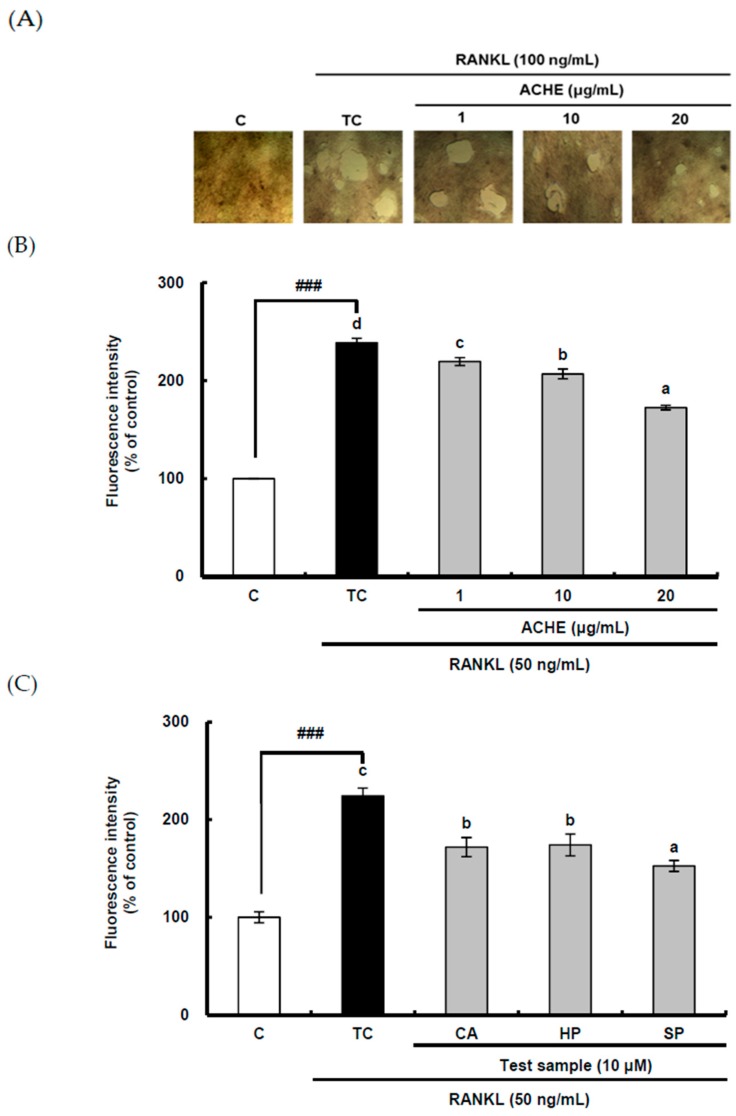
Inhibitory effect of hydroethanolic extract of *A. capillaris* and its bioactive compounds on bone resorption. (**A**) The visualized absorbed area with ACHE treatment; (**B**) The fluorescence intensity of the absorbed area with treatment with ACHE and (**C**) three bioactive compounds. A bone resorption assay was performed using a commercial assay kit. RAW 264.7 cells were exposed to RANKL (100 ng/mL) for 5 days in the absence and presence of ACHE or three compounds. The absorbed areas on each plate were visualized at 200-fold magnification under light microscopy. Fluorescence intensity was measured at an excitation wavelength of 485 nm and an emission wavelength of 535 nm using a fluorometric plate reader. Data are expressed as percentages of the values of untreated cells (means ± standard deviations, *n* = 3). Different corresponding letters indicate significant differences at *p* < 0.05 by Duncan’s test. ^###^ *p* < 0.001 vs. C. C: control, which was not treated; TC: treated control, which was treated with RANKL; CA: chlorogenic acid; HP: hyperoside; SP: scoparone.

The inhibitory effect of ACHE on bone resorption was determined in RANKL-differentiated osteoclasts cultured for 5 days on well plates coated with fluoresceinamine-labeled chondroitin sulfate (FACS) and calcium phosphate (CaP) using a bone resorption assay kit (CosMo Bio, Tokyo, Japan). The FACS bound to CaP was released from the CaP layer into the medium by osteoclastic resorption. Therefore, bone resorption activity is proportional to fluorescence intensity of FACS in the medium. The resorbed area on the plates was visualized under 200-fold magnification under light microscopy. RANKL treatment markedly increased the number and size of bone resorption areas (Figure 4A). Consistently, the bone resorption activity was significantly (*p* < 0.001) enhanced by RANKL (Figure 4B). In contrast, ACHE at 1–20 μg/mL dose-dependently alleviated bone resorption of osteocla sts.

Bone resorption can be achieved by multi-nucleated osteoclasts after the differentiation process. Because the strong inhibitory effect on osteoclastic differentiation was found in three compounds such as chlorogenic acid, hyperoside, and scoparone, these three bioactive compounds were used for bone resorption assay. To determine which of the three compounds of ACHE with potential anti-osteoclastic activity could be related with the inhibitory activity of ACHE on bone resorption of osteoclasts, the bone resorption assay was carried out using a bone resorption assay kit. Though all of three compounds showed the inhibition effect on bone resorption (Figure 4C), the strongest inhibition effect was observed in scoparone. These data imply that scoparone as well as chlogenic acid and hyperoside may be major components of ACHE, contributing to its inhibitory activity on bone resorption of osteoclasts.

### 2.4. Suppressive Effect of ACHE and Three Bioactive Compounds on the Acidification by Osteoclasts

**Figure 5:**
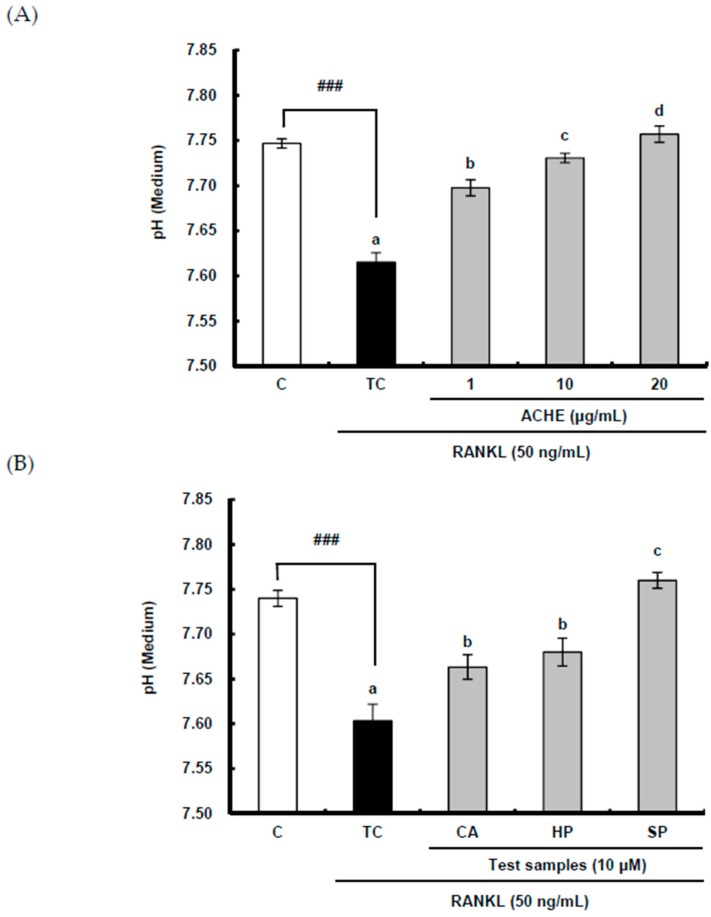
(**A**) Inhibitory effect of hydroethanolic extract of *A. capillaris* and (**B**) three bioactive compounds on acidification. RAW 264.7 cells were exposed to RANKL (50 ng/mL) for 5 days in the absence and presence of ACHE. After 5 days, the pH of the differentiated medium was measured using a pH meter. Data are expressed as percentages of the values of untreated cells (means ± standard deviations, *n* = 3). Different corresponding letters indicate significant differences at *p* < 0.05 by Duncan’s test. ^##^ *p* < 0.01, ^###^ *p* < 0.01 vs. C. C: control, which was not treated; TC: treated control, which was treated with RANKL; CA: chlorogenic acid; HP: hyperoside; SP: scoparone.

The effect of ACE on acidification was examined by measuring pH since the acidic pH is essential for bone resorption by ostaoclasts. RANKL significantly (*p* < 0.001) diminished the pH of a culture medium as compared to the control group, but ACHE at 1–20 μg/mL reversed this decrease and further augmented pH in a dose-dependent manner (Figure 5A). In addition, the enhancing effect of three compounds on pH is clearly shown in Figure 5B. Furthermore, scoparone markedly increased pH as compared to chlorogenic acid and hyperoside, which is in line with the result of their suppressive effects on bone resorption.

### 2.5. Down-Regulating Effect of ACHE and Three Bioactive Compounds on TRAF6 Expression and Its Binding to V-ATPase

**Figure 6:**
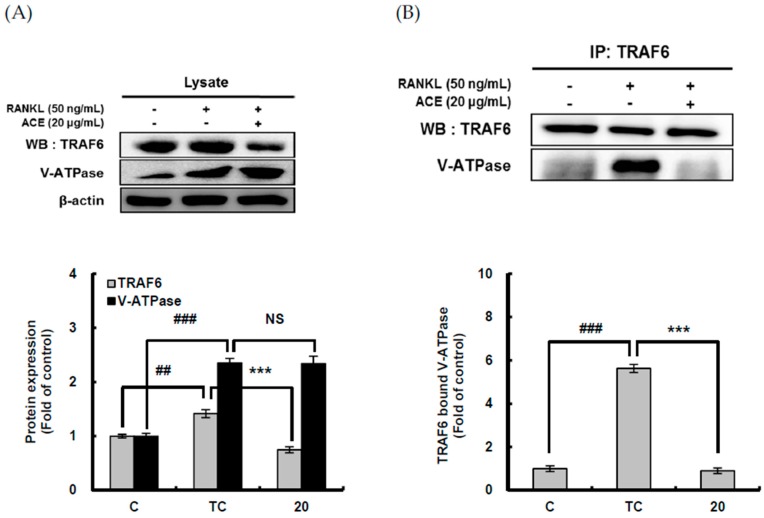

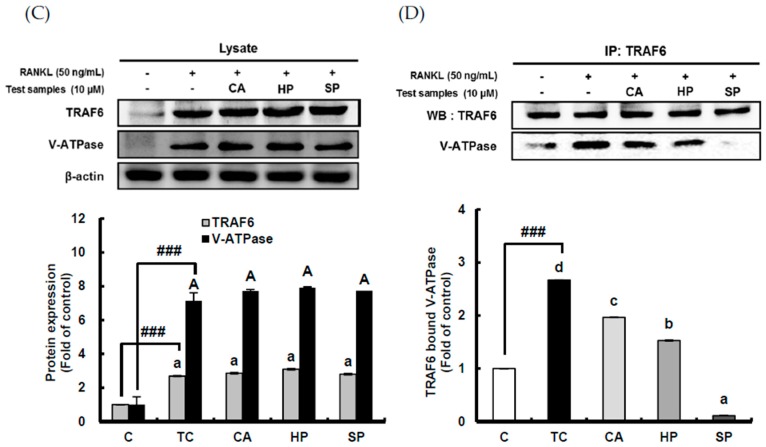
Suppressive effect of hydroethanolic extract of *A. capillaris* and three bioactive compounds on tumor necrosis factor receptor-associated factor 6 (TRAF6) and vacuolar H^+^-adenosine triphosphatase (V-ATPase) expression and their interaction. (**A**) The effect of *A. capillaris* hydroethanolic extract (ACHE) on TRAF6 and V-ATPase expression and (**B**) their interaction. (**C**) The effect of three bioactive compounds on TRAF6 and V-ATPase expression and (**D**) their interaction. RAW 264.7 cells were exposed to RANKL (50 ng/mL) for 5 days in the absence and presence of ACHE (20 μg/mL) or three bioactive compounds (10 μM). The cells were lysed and cell lysates were incubated with TRAF6 or V-ATPase antibody and protein A beads. After washing the beads, precipitated proteins and cell lysates were separated by sodium dodecyl sulfate-polyacrylamide gel electrophoresis (SDS-PAGE) and immunoblotted for the indicated proteins using specific antibodies. Data are expressed as percentages of the values of untreated cells (means ± standard deviations, *n* = 3). ^##^ *p* < 0.01, ^###^ *p* < 0.001 vs. C; *** *p* < 0.001 vs. TC. C: control, which was not treated; TC: treated control, which was treated with RANKL; CA: chlorogenic acid; HP: hyperoside; SP: scoparone; NS: not significant.

RANKL binding to RANK localized on the membrane of precursor cells may recruit TRAF6 to induce osteoclast differentiation and to enhance bone resorption. For efficient bone resorption by osteoclasts, acidification is required and the primary mechanism responsible for this acidification is V-ATPase which transports H^+^ to extracellular resorption lacunae [7]. The interaction of V-ATPase with TRAF6 recruited by the receptor activator of nuclear factor-κB (RANK) results in its activation [8]. Therefore, the effect of ACHE on expression of TRAF6 and V-ATPase and their interaction was investigated using western blot analysis and immunoprecipitation. RANKL significantly (*p* < 0.001) enhanced the expression of both TRAF6 and V-ATPase (Figure 6A). However, ACHE at 20 μg/mL significantly (*p* < 0.001) diminished the TRAF6 expression enhanced by RANKL, but showed no effect on V-ATPase expression increased by RANKL. Furthermore, to determine whether V-ATPase binds with TRAF6 for its activation, the association of TRAF6 with V-ATPase was investigated. In anti-TRAF6 immunoprecipitates, RANKL significantly (*p* < 0.001) augmented V-ATPase bound to TRAF6, and ACHE at 20 μg/mL significantly (*p* < 0.001) abrogated this increased V-ATPase (Figure 6B).

Which of the three compounds contributed to the inhibitory effect on expression of TRAF6 and its interaction with V-ATPase was examined. According to Figure 6C, the significant difference in the protein level of TRAF6 and V-ATPase was not found among three compounds (Figure 6C). In addition, in anti-V-ATPase immunoprecipitates, RANKL significantly (*p* < 0.001) induced the interaction V-ATPase with TRAF6. The most potent inhibition effect on interaction V-ATPase with TRAF6 was observed in scoparone, followed by hyperoside and chlorogenic acid (Figure 6D). This data is consistent with the result of the inhibitory effect of three compounds from *A. capillaris* on acidification by osteoclasts. Consequently, these results strongly suggest that scoparone as well as chlorogenic acid and hyperoside are responsible compounds of ACE for attenuating the association of V-ATPase with TRAF6.

## 3. Discussion

The osteoclast responsible for bone resorption is a multinucleated cell differentiated from mononuclear precursors including monocytes and macrophages [3]. The low level of nontoxic amount of reactive oxygen species (ROS) with several growth factors and cytokines is induced by the RANKL binding to the receptors [5]. This increase in ROS may play an important role as a secondary messenger in RANKL-mediated signaling pathways for osteoclast differentiation [6]. Previous reports have demonstrated that potent flavonoid antioxidants ameliorating osteoclastic differentiation have been also found in genistein [22], luteolin [23], baicalein [24], epigallocatechin-3-gallate [25], and fisetin [4]. Our previous study confirmed that scopoletin with strong cellular antioxidant capacity reduces ROS production as superoxide anions to suppress osteoclastic differentiation from RAW 264.7 cells [26]. In addition, the potent antioxidant activity of *A. capillaris* was recently reported [13,27]. Thus, these reports have led us to investigate the inhibitory effect of *A. capillaris* on RANKL-induced osteoclast differentiation.

In the present study, the anti-osteoclastic effect of A. capillaris was confirmed by measuring the number and TRAP activity of osteoclasts, which is consistent with the recent study reporting the suppressive effect of *A. capillaris* on osteoclast differentiation [20]. This anti-osteoclastic effect of *A. capillaris* may be attributed to three bioactive compounds such as chlorogenic acid, hyperoside, and scoparone because the potent suppressive effect on osteoclast differentiation was found in them compared to caffeic acid, isoquercitrin, and isochlorogenic acid. Recently, it has been reported that scoprone can attenuate RANKL-induced osteoclastic differentiation through suppressing ROS production and scavenging [21] and chlorogenic acid can inhibit RANKL-induced osteoclast differentiation [28], implying that the anti-osteoclastic effect of *A. capillaris* may be attributed to the antioxidant activity of three bioactive compounds including chlorogenic acid, hyperoside, and scoparone.

Excessive bone resorption by osteoclasts can result in osteoporosis in older post-menopausal women. The inhibitory effects of natural polyphenols including silibinin and phloretin on bone resorption have been also reported [29,30]. To check the possibility of *A. capillaris* in the treatment of osteoporosis through regulating bone resorption, the suppressive effect of *A. capillaris* on bone resorption by osteoclasts was observed in the current study. For efficient bone resorption, the solubilization of bone mineral and the hydrolysis of organic bone matrix by enzymes are required, which depends on the pH of the environment. The result that *A. capillaris* significantly augmented pH lowered by RANKL treatment appears to be associated with decreased bone resorption. According to Figure 4 and 5, the inhibitory effect of scoparone on bone resorption and acidification was more potent than that of chlorogenic acid and hyperoside. In addition, the inhibitory effect of chlorogenic acid on bone resorption was reported in the previous study [28]. Therefore, these data support that scoparone as well as chlorogenic acid and hyperoside may be major active components of *A. capillaris*, attenuating bone resorption of osteoclast through controlling acidification.

The primary cellular protein of the acidification by osteoclasts is known as V-ATPase which hydrolyzes ATP to produce protons [7]. In addition, the interaction of V-ATPase with TRAF6 is essential for its activation [8]. The present study revealed that *A. capillaris* markedly alleviated TRAF6 expression and the association of TRAF6 with V-ATPase compared to RANKL treatment. In addition, although three bioactive compounds including chlorogenic acid, hyperoside, and scoparone displayed the potent inhibitory effect on bone resorption in comparison with caffeic acid, isoquercitrin, and isochlorogenic acid, they did not significantly affect the expression of either TRAF6 or V-ATPase. In contrast, they vigorously interrupted the association of V-ATPase with TRAF6 compared to RANKL treatment even though the underlying mechanism remains unclear. The possible explanation for the reduced bone resorption of *A. capillaris* due to suppresssed acidification may be the reduced osteoclast differentiation and/or the attenuated interaction of V-ATPase with TRAF6. Accordingly, this finding indicates that scoparone, as well as chlorogenic acid and hyperoside, may be partially responsible for the inhibitory effect of *A. capillaris* on bone resorption through disrupting the association of V-ATPase with TRAF6.

## 4. Materials and Methods

### 4.1. Reagents and Cell Culture Materials

Neocuproine, Dulbecco’s modified Eagle’s medium (DMEM), minimum essential medium alpha medium (α-MEM), fetal bovine serum (FBS), 2,5-diphenyltetrazolium (MTT), Triton X-100, RANKL, sodium tartrate, *p*-nitro-phenylphosphate (PNPP), fluoresceinamine-labeled chondroitin sulfate (FACS), phosphate-buffered saline (PBS, pH 7.4), ethylenediaminetetraacetic acid (EDTA), dithiothreitol (DTT), phenylmethanesulfonyl fluoride (PMSF), dimethylsulfoxide (DMSO), chlorogenic acid, caffeic acid, isoquercitrin, and isochlorogenic acid A were purchased from Sigma-Aldrich (St. Louis, MO, USA). Hyeroside was purchased from Hwz Analytic GmbH (Ruelzheim, Germany). Scoparone was obtained from Phytolab GmbH&Co (Vestenbergreuth, Germany). Antibodies and protein A-agarose beads were purchased from Santa Cruz Biotechnology (Santa Cruz, CA, USA), including anti-TRAF6 (sc-7221), V-ATPase (sc-20946), and β-actin (sc-130656). A bone resorption assay kit was purchased from CosMo Bio Co., Ltd. (Tokyo, Japan). MC3T3-E1 subclone 4 and RAW 264.7 cells were obtained from the American Type Culture Collection (ATCC, Rockville, MD, USA).

### 4.2. Preparation of A. capillaris Hydroethanolic Extract (ACHE)

The aerial parts of *A. capillaris* Thunb. were purchased at Kyoungdong market, an herbal medicine market in Seoul, and taxonomically identified by Young-Ho Kim who is a professor in the College of Pharmacy, Chungnam National University, Daejeon, Republic of Korea. A voucher specimen (CNU-10103) was deposited at the Herbarium of the College of Pharmacy, Chungnam National University. The plants were cut in 1-cm lengths and were extracted with a 6-fold volume of 50% ethanol at 60 °C for 6 h. Then, a second extraction was done with a 5-fold volume of 50% ethanol at 50 °C for 16 h. After the pooled extraction solution was concentrated under a vacuum and spray-dried, it was kept at −20 °C until used.

### 4.3. High Performance Liquid Chromatography (HPLC) Analysis

The HPLC analysis was carried out using a HP Agilient HPLC system (Santa Clara, CA, USA) with a solvent delivery unit, an online degasser, a column oven, an autosampler and a photodiode array (PDA) detector. The analytical column was C18 (21.5 × 300 mm, particle size 10 μM, Tosoh, Tokyo, Japan) maintained at 40 °C. The mobile phases were distilled water (A) and phosphoric acid in 60% acetonitrile (pH 2.4, B). The gradient flow rate was composed of 0%–10% B for 0–5 min, 10%–50% B for 5–30 min, and 50%–100% B for 30–35 min. The flow rates and injection volumes were 1.0 mL/min and 10 μL, respectively. The detection wavelength was 255 nm for hyperoside and isoquercitrin, 320 nm for chlorogenic acid, caffeic acid, and isochlorogenic acid A, and 340 nm for scoparone. Six pure compounds such as chlorogenic acid, caffeic acid, hyperoside, isoquercitrin, isochlorogenic acid A, and scoparone, were used as chemical markers. The identification of the compounds was performed by comparison of the retention time and UV spectrum.

### 4.4. Cell Cytotoxicity by MTT Assay

RAW 264.7 cells were plated in 24-well plates at a density of 2 × 10^4^ cells/mL in triplicate. Cells were treated with RANKL (50 ng/mL) and increasing concentrations of ACHE (1–50 μg/mL), or six compounds (1–20 µM) were added to DMEM supplemented with 10% (*v*/*v*) FBS and 1% (*v*/*v*) antibiotics. After 5 days, MTT reagent was added to each well. The plate was incubated at 37 °C for 1 h. After removing the medium, the plate was washed twice with PBS. DMSO was then added to dissolve the intracellular insoluble formazan. The absorbance was measured at 570 nm using an enzyme-linked immunosorbent assay (ELISA) reader (Tecan, Salzburg, Austria), and the percentage proliferation was calculated.

### 4.5. TRAP Staining and Activity

RAW 264.7 cells were plated in 96-well plates at a density of 1 × 10^4^ cells/well. The cells were treated with RANKL (50 ng/mL) and ACHE (1–20 μg/mL), or six compounds (10 µM) were added to DMEM medium supplemented with 10% (*v*/*v*) FBS and 1% (*v*/*v*) antibiotics. The medium was changed every 2 days. After 5 days, the cells were fixed in 3.5% formalin for 10 min and stained with an acid phosphatase kit. The multinucleated osteoclast cells which were stanined with TRAP, were observed at 200-fold magnification with a light microscopy. For measuring TRAP activity, the cells was washed twice with ice-cold PBS and fixed in 3.5% formaldehyde and ethanol/acetone (1:1) for 10 and 1 min, respectively. The dried cells were then incubated in 50 mM citrate buffer (pH 4.5) supplemented with 10 mM sodium tartrate and 6 mM PNPP for 40 min. After the mixtures were added to the new wells with an equal volume of 0.1 N NaOH, the absorbance was determined at 405 nm using an ELISA reader.

### 4.6. Bone Resorption Assay

A bone resorption assay kit (CosMo Bio, Tokyo, Japan) was used for bone resorption assay. In this assay, phenol red-free DMEM was used to avoid the interruption of fluorescence measurement due to phenol red. RAW 264.7 cells were seeded in FACS and CaP-coated 24-well plates (1 × 10^4^ cells/well) containing phenol red-free DMEM plus 10% FBS, and the medium was replaced with test sample (1 to 20 µg/mL ACHE or 10 µM six compounds) in DMEM containing 50 ng/mL RANKL. After 5 days, 100 μL of the medium and 50 μL resorption assay buffer (catalogue number CSR-BRA-B1, CosMo Bio Co. Ltd) were transferred into the wells of a 96-well plate and mixed for 3 min under dark conditions. A fluorometric plate reader (Tecan GENios, Salzburg, Austria) was used to measure the fluorescence at an excitation of 485 nm and an emission of 535 nm. After washing the cells with 5% sodium hypochlorite, the resorbed areas on the plate were visualized at 200-fold magnification under light microscopy.

### 4.7. pH Measurement

RAW 264.7 cells were seeded in 60 mm dishes (1 × 10^4^ cells/dish) containing DMEM plus 10% FBS, and the medium was replaced with test sample (1–20 μg/mL ACHE or 10 μM of six compounds) in differentiation medium. The differentiation medium was changed every 2 days. After 5 days, the pH of the differentiated media was measured using a pH meter (Thermo Scientific, Hudson, NH, USA).

### 4.8. Immunoprecipitation and Immunoblotting

Immunoprecipitation was performed according to the method of Cho et al. with a slight modification [31]. RAW 264.7 cells were plated in 6-well plates a density of 1 × 10^4^ cells/well. The cells were treated with RANKL (50 ng/mL) and ACHE (1–20 μg/mL) or six compounds (10 μM) were added to DMEM medium supplemented with 10% (*v*/*v*) FBS and 1% (*v*/*v*) antibiotics. The medium was changed every 2 days. After 5 days, the cell were harvested using a cell scraper and centrifuged at 7500× *g* for 20 min. The cell pellets were lysed in NaCl EDTA Tris-nonyl phenoxypolyethoxylethanol (NP)-40 lysis (NET-NL) buffer (1 M Tris at pH 7.5, 0.5 M EDTA, 1 M NaCl, 1 M DTT, 10% NP-40, 0.1 M PMSF, 10 mg/mL bovine serum albumin (BSA), and protease inhibitor cocktail). The cell lysates were immunoprecipitated with 2 µg of TRAF6 and V-ATPase antibodies, and protein A–agarose beads by overnight incubation. The immune complexes were then washed three times with NaCl EDTA Tris-NP-40 washing (NET-NW) buffer (1 M Tris at pH 7.5, 0.5 M EDTA, 1 M NaCl, 1 M DTT, 10% NP-40, and 0.1 M PMSF) and centrifuged at 570× *g* for 30 s. The immunoprecipitated proteins were mixed with sample loading buffer, resolved by 8%–10% SDS-PAGE, and immunoblotted using anti-TRAF6, anti-V-ATPase, and anti-β-actin antibodies. The proteins on nirocellulose membranes were detected with a chemiluminescence kit (Intron Biotechnology, Seoul, Korea) and analyzed using a LAS4000 chemiluminescent image analyser (Fuji, Tokyo, Japan).

### 4.9. Statistical Analysis

All data are presented as mean ± standard deviation. Statistical analysis were done by the statistical package for the social sciences (SPSS, Chicago, IL, USA) program. Student’s t-test was used the parameters between two groups. One-way analysis of variance (ANOVA) and Duncan’s test were used to compare the parameters among more than three groups and a *p* < 0.05 was considered statistically significant.

## 5. Conclusions

The current report demonstrates that ACHE, an *A. capillaris* hydroethanolic extract, attenuated osteoclastic effect including RANKL-induced osteoclast differentiation and bone resorption activity; the responsible compounds for this effect were chlorogenic acid, hyperoside, and scoparone. The blockage of bone resorption by *A. capillaris* was in part mediated by reducing acidification through down-regulating the interaction of V-ATPase with TRAF6 due to scoparone as well as chlorogenic acid and hyperoside. For these reasons, *A. capillaris* extract may have a high potential as an important bioactive resource of functional food for the prevention or treatment of bone diseases caused by bone loss. However, additional studies using in vivo models and clinical trials are required.

## Figures and Tables

**Table 1:** The content of six marker compounds of hydroethanolic extract of *A. capillaris*. (*n* = 3).

Compounds	Content ^(1)^ (mg/g)
Chlorogenic acid	38.526 ± 0.927
Caffeic acid	0.515 ± 0.048
Hyperoside	4.072 ± 0.220
Isoquercitrin	3.031 ± 0.148
Isochlorogenic acid	13.898 ± 0.667
Scoparone	6.589 ± 0.193

^(1)^ Each value is expressed as mean ± standard deviation in triplicate experiments.
